# Supplementary material for: Multi-Omics of Campylobacter jejuni Growth in Chicken Exudate Reveals Molecular Remodelling Associated with Altered Virulence and Survival Phenotypes
Source: Microorganisms. 2024 Apr 25;12(5):860. doi: 10.3390/microorganisms12050860 (PMC11123243; doi:10.3390/microorganisms12050860)
Supplement: Supplementary file 1 [file microorganisms-12-00860-s001.zip › Supplementary Figures S1_S6.pdf]

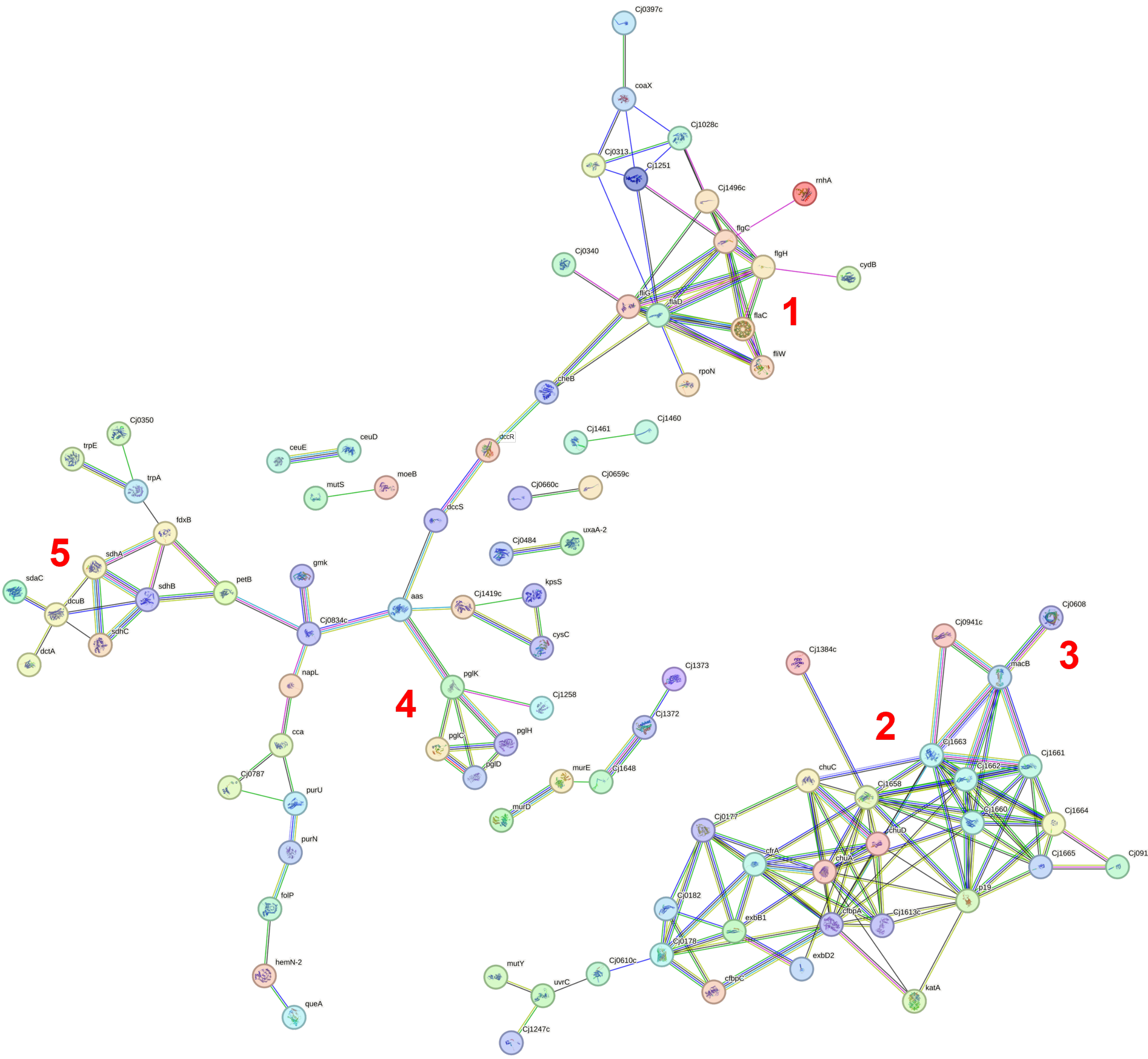

Supplementary Figure S1A. STRINGdb analysis of proteins significantly elevated in abundance in *C. jejuni* grown in MH medium with 5% CE compared with MH only control. Enriched functions included (1) flagellar motility, (2) iron uptake and antioxidants, (3) antibiotic resistance, (4) the *pgl*-encoded protein glycosylation system and (5) nutrient transporters and metabolism. Minimum required interaction score was set to confidence 0.700, cut-off for inclusion was >+1.5 fold-change; 181 edges observed, 74 expected with PPI enrichment *p*-value <1.0e-16.

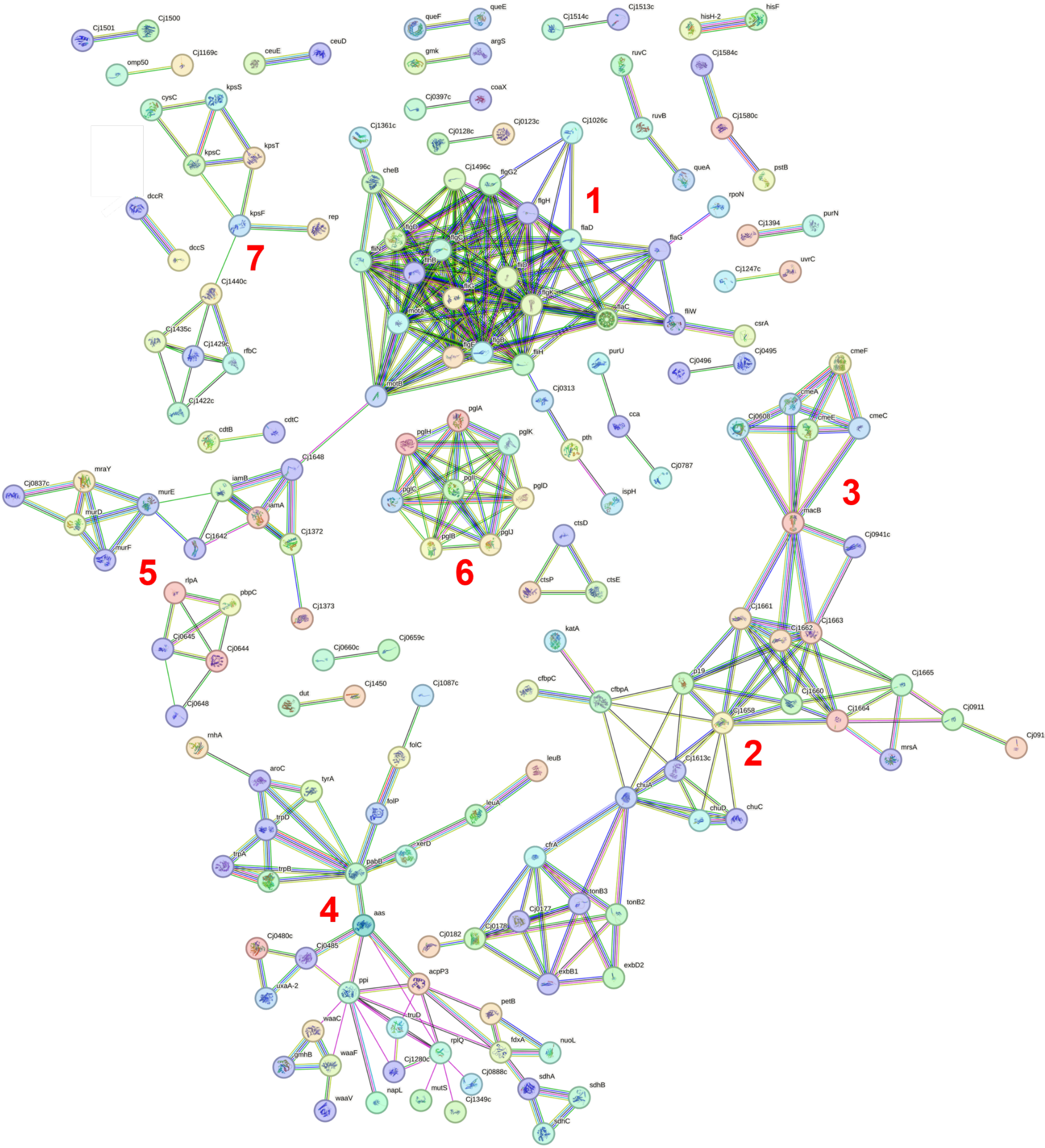

Supplementary Figure S1B. STRINGdb analysis of proteins significantly elevated in abundance in *C. jejuni* grown in 100% CE compared with MH only control. Enriched functions included (1) flagellar motility, (2) iron uptake and antioxidants, (3) antibiotic resistance, (4) nutrient transport and metabolism, (5) peptidoglycan structure and cell shape, (6) the *pgl*-encoded protein glycosylation system and (7) lipooligosaccharide. Minimum required interaction score was set to confidence 0.700, cut-off for inclusion was >+1.5 fold-change; 381 edges observed, 182 expected with PPI enrichment *p*-value <1.0e-16.

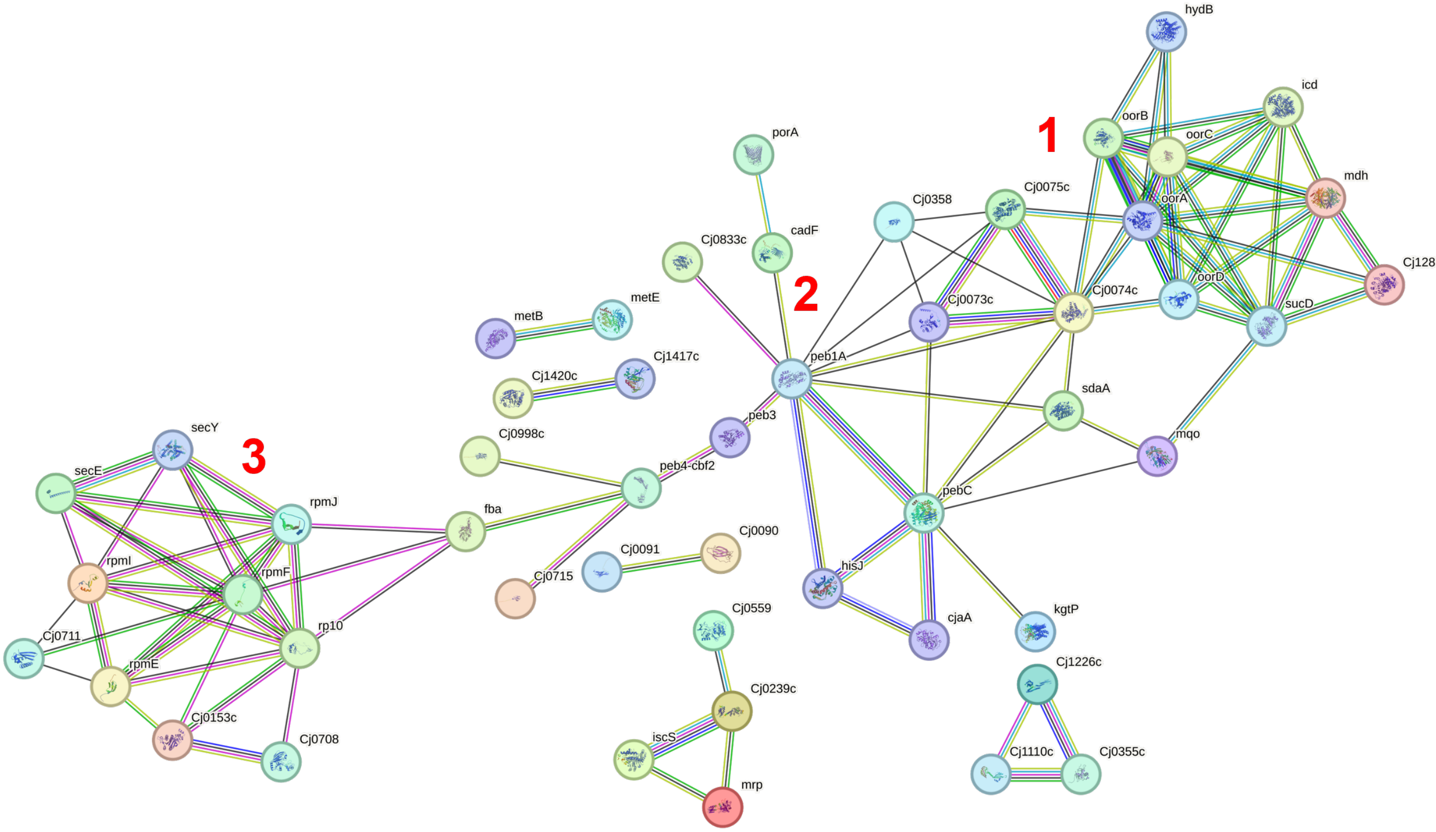

Supplementary Figure S1C. STRINGdb analysis of proteins significantly reduced in abundance in *C. jejuni* grown in MH medium with 5% CE compared with MH only control. Enriched functions included (1) metabolism, (2) virulence and antigenicity and (3) translation and protein secretion. Minimum required interaction score was set to confidence 0.700, cut-off for inclusion was <-1.5 fold-change (<0.67-fold); 104 edges observed, 52 expected with PPI enrichment *p*-value =8.9e-11.

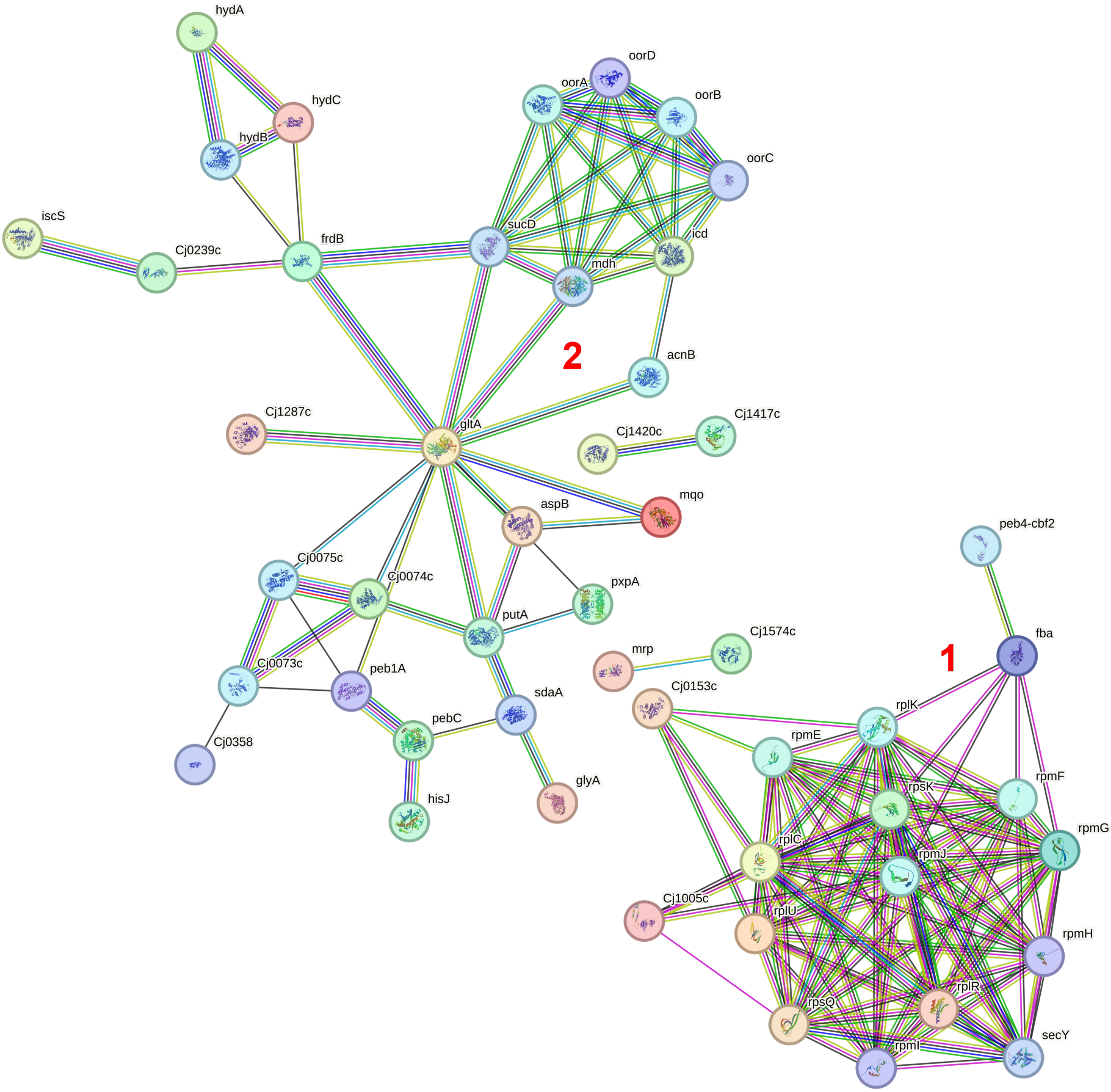

Supplementary Figure S1D. STRINGdb analysis of proteins significantly reduced in abundance in *C. jejuni* grown in 100% CE compared with MH only control. Enriched functions included (1) translation and (2) metabolism. Minimum required interaction score was set to confidence 0.700, cut-off for inclusion was <-1.5 fold-change (<0.67-fold); 150 edges observed, 96 expected with PPI enrichment *p*-value =2.7e-7.

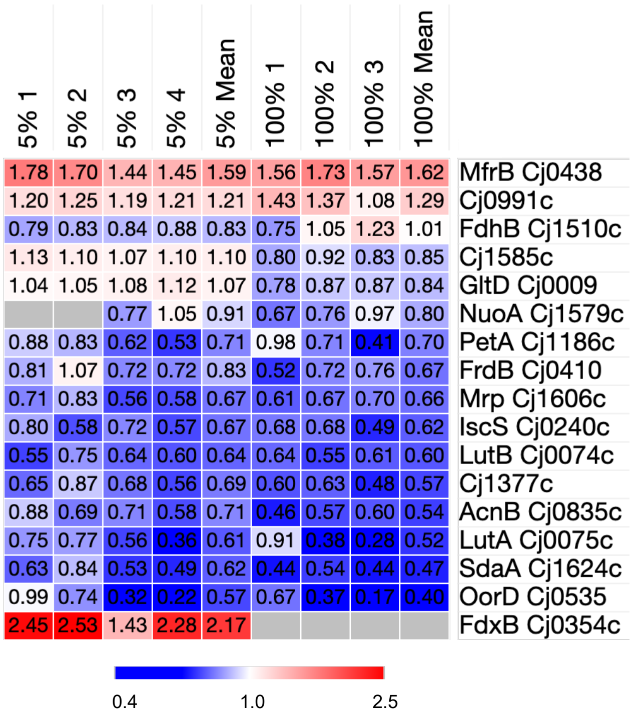

Supplementary Figure S2. Elevated abundances of iron acquisition proteins correlate with reduced abundances of proteins containing Fe-S clusters. Heat map showing replicate (MH+5% CE [left; *n*=4] and 100% CE [right; *n*=3] compared with MH only control) and mean *n*-fold data for proteins containing Fe-S clusters.

5% 1	5% 2	5% 3	5% 4	5% Mean	100% 1	100% 2	100% 3	100% Mean	
1.63	1.50	1.20	1.95	1.57	2.27	2.74	2.79	2.60	AnsA AnsB Cj0029
2.22	1.80	1.18	1.11	1.58	2.17	3.99	1.56	2.57	PetB Cj1185c
1.10	1.13	2.47	1.23	1.48	2.33	2.86	1.30	2.16	TrpD (TrpGD) Cj0346
1.83	1.66	1.39	1.63	1.63	1.59	2.35	1.91	1.95	MfrA SdhA Cj0437
1.18	1.23	1.70	1.29	1.35	1.97	1.81	1.99	1.92	Cj1513c
3.50	1.71	1.39	1.05	1.91	1.83	2.26	1.42	1.83	CioB cydB Cj0082
1.11	1.60	1.42	2.15	1.57	1.89	1.49	2.07	1.81	TrpA Cj0349
1.01	1.67	1.13	1.41	1.30	2.30	1.59	1.49	1.80	TrpB Cj0348
1.39	1.42	1.49	2.21	1.63	1.76	1.76	1.62	1.71	NapL Cj0784
0.95	1.28	1.13	1.14	1.12	1.89	2.05	1.14	1.69	FdhM Cj1514c
1.32	1.35	1.32	1.32	1.33	1.98	1.51	1.57	1.69	GapA Cj1403c
1.39	1.44	1.81	1.45	1.52	2.33	1.44	1.29	1.69	MfrC SdhC Cj0439
1.78	1.70	1.44	1.45	1.59	1.56	1.73	1.57	1.62	MfrB SdhB Cj0438
1.10	1.37	1.32	1.27	1.26	1.72	1.47	1.02	1.40	NapB Cj0783
1.08	2.74	1.27	1.36	1.61	1.20	1.29	1.47	1.32	TrpE Cj0345
1.45	1.53	1.32	1.35	1.41	1.03	1.26	1.55	1.28	NapD Cj0785
1.28				1.28			1.27	1.27	CcoQ Cj1488c
1.36	1.21	1.32	1.24	1.29	1.51	1.26	0.98	1.25	GpmI Pgm Cj0434
1.26	1.21	0.61	0.74	1.24		1.23		1.23	CcoN Cj1490c
1.06	1.07	0.98	1.04	1.04	1.40	0.85	1.10	1.12	NapG Cj0781
0.98	0.92	1.00	1.00	0.98	1.32	0.97	1.05	1.11	Fbp Cj0840c
0.99	1.11	1.08	1.14	1.08	1.15	0.97	1.01	1.04	AspA Cj0087
1.26	1.15	1.31	1.17	1.22	0.96	0.98	1.15	1.03	NapA Cj0780
0.79	0.83	0.84	0.88	0.83	0.75	1.05	1.23	1.01	FdhB Cj1510c
0.95	0.75	0.93	0.73	0.84	1.43	0.78	0.81	1.01	TpiA Cj1401c
1.11	1.02	1.18	1.04	1.09	0.82	1.34	0.84	1.00	Pyk Cj0392c
0.87	0.94	0.89	0.93	0.91	1.13	0.95	0.92	1.00	PycB Cj0933c
0.81	0.86	0.86	0.84	0.84	1.01	0.91	1.06	0.99	FdhD Cj1508c
0.97	0.98	0.95	1.06	0.99	1.01	0.87	1.06	0.98	PetC Cj1184c
0.85	0.86	0.89	0.89	0.87	0.79	0.97	0.99	0.92	GltB Cj0007
0.93	0.92	0.91	0.92	0.92	0.81	0.86	1.08	0.91	Pgk Cj1402c
0.77	0.83	1.02	0.94	0.89	0.91	0.81	0.99	0.90	FrdC Cj0408
0.96	0.93	1.08	1.03	1.00	0.79	0.95	0.84	0.86	CcoO Cj1489c
1.10	1.03	1.11	1.06	1.08	0.94	0.78	0.86	0.86	Tal Cj0281c
0.84	0.89	0.81	0.92	0.87	0.80	0.86	0.88	0.85	FdhA Cj1511c
1.07	1.12	1.09	1.16	1.11	1.02	0.78	0.74	0.84	FrdA Cj0409
1.04	1.05	1.08	1.12	1.07	0.78	0.87	0.87	0.84	GltD Cj0009
0.94	0.90	0.92	0.88	0.91	0.81	0.87	0.78	0.82	PycA Cj1037c
1.45	1.41	1.31	1.50	1.42		0.86	0.73	0.80	NapH Cj0782
0.68	0.65	0.67	0.59	0.65	0.56	0.55	1.24	0.79	CioA cydA Cj0081
0.97	0.90	1.00	0.72	0.90	0.81	0.77	0.76	0.78	SucC Cj0533
0.99	0.98	1.05	0.99	1.00	0.81	0.70	0.81	0.77	PckA Cj0932c
0.80	0.90	0.84	0.65	0.80	0.55	1.10	0.53	0.73	CcoP Cj1487c
0.88	0.83	0.62	0.53	0.71	0.98	0.71	0.41	0.70	PetA Cj1186c
0.75	0.66	0.63	0.60	0.66	0.62	0.64	0.75	0.67	Mqo Cj0393c
0.81	1.07	0.72	0.72	0.83	0.52	0.72	0.76	0.67	FrdB Cj0410
0.55	0.58	0.64	0.74	0.63	0.68	0.69	0.63	0.67	Fba FdaC Cj0597
		0.47	0.51	0.49	0.50	0.66	0.80	0.65	FdhC Cj1509c
0.96	0.93	0.88	0.68	0.86	0.62	0.62	0.69	0.64	GltA Cj1682c
0.88	0.88	1.39	1.37	1.38		0.64	0.59	0.61	TrpF Cj0347
0.23	0.87	0.75	0.66	0.63	0.67	0.50	0.67	0.61	Icd Cj0531
0.55	0.75	0.64	0.60	0.64	0.64	0.55	0.61	0.60	LutB Cj0074c
0.71	0.83	0.64	0.57	0.69	0.74	0.53	0.45	0.57	PutA Cj1503c
0.64	0.47	0.55	0.40	0.52	0.65	0.56	0.45	0.55	OorA Cj0536
0.88	0.69	0.71	0.58	0.71	0.46	0.57	0.60	0.54	AcnB Cj0835c
0.87	0.57	0.68	0.36	0.62	0.57	0.68	0.36	0.53	OorB Cj0537
0.26	0.21	0.82	0.55	0.46	0.55	0.55	0.49	0.53	LutC Cj0073c
0.75	0.77	0.56	0.36	0.61	0.91	0.38	0.28	0.52	LutA Cj0075c
0.71	0.53	0.58	0.41	0.56	0.56	0.50	0.37	0.48	SucD Cj0534
0.29	0.91	0.82	0.60	0.66	0.29	0.59	0.55	0.48	Mdh Cj0532
0.63	0.84	0.53	0.49	0.62	0.44	0.54	0.44	0.47	SdaA Cj1624c
1.12	0.28	0.72	0.42	0.63	0.41	0.61	0.37	0.46	OorC Cj0538
0.99	0.74	0.32	0.22	0.57	0.67	0.37	0.17	0.40	OorD Cj0535

0.4 1.0 2.5

Supplementary Figure S3. Growth in CE alters *C. jejuni* proteins associated with metabolism. Heat maps showing replicate (MH+5% CE [left; *n*=4] and 100% CE [right; *n*=3] compared with MH only control) and mean *n*-fold data for proteins involved in metabolism.

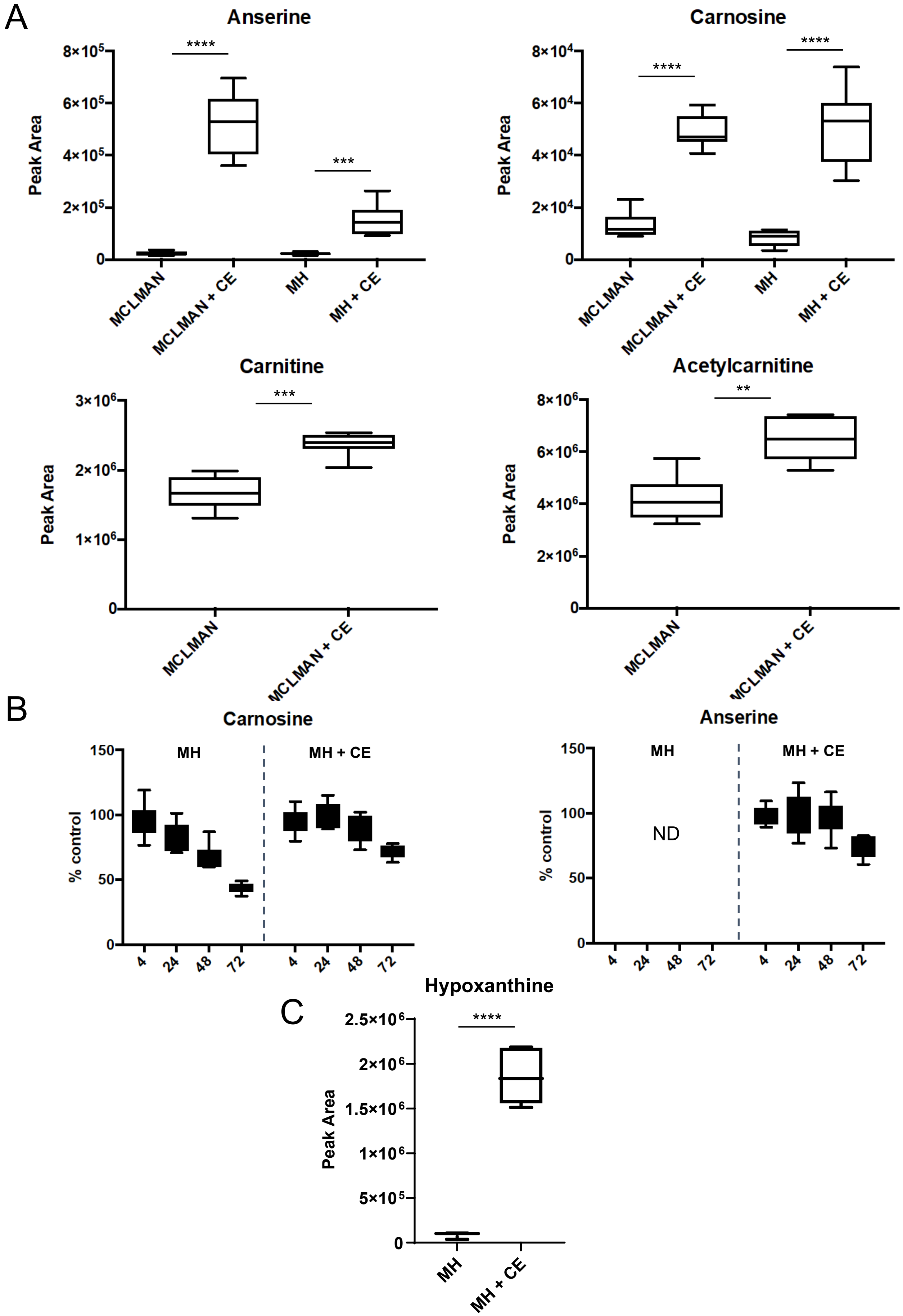

Supplementary Figure S4. Evidence of *C. jejuni* uptake of chicken meat-associated compounds carnosine and anserine. (**A**) Intracellular metabolite abundances as determined by targeted LC-MS/MS. (**B**) Culture supernatant levels as determined by targeted LC-MS/MS, box and whisker plots based on peak area values determined as a % of the corresponding non-inoculated control (MH and MH+5%CE) at each time-point. (**C**) Intracellular abundance of hypoxanthine. **, $p < 0.01$; ***, $p < 0.001$; ****, $p < 0.0001$); ND, not detected.

A

5% 1	5% 2	5% 3	5% 4	5% Mean	100% 1	100% 2	100% 3	100% Mean	
1.17	1.72	1.50	1.64	1.51	1.71	1.76	2.06	1.84	CheB' Cj0924c
1.19	1.14	1.23	1.29	1.21	1.80	1.08	1.06	1.31	Tlp6 Cj0448c
0.97	0.97	1.05	1.02	1.00	1.19	0.97	0.95	1.04	CheR Cj0923c
0.71	0.79	0.75	0.82	0.77	1.28	0.90	0.77	0.98	Tlp10 Cj0019c
0.86	0.86	0.86	0.89	0.87	0.82	0.94	1.13	0.96	Tlp2 Cj0144
1.08	1.01	1.13	0.95	1.04	0.86	1.35	0.64	0.95	CheY Cj1118c
1.06	1.11	1.11	1.13	1.10	1.19	0.74	0.87	0.93	Cj0700
0.68	0.73	0.74	0.74	0.73	0.86	0.87	1.05	0.93	Tlp4 Cj0262c
1.08	1.02	1.12	1.03	1.06	0.87	1.15	0.73	0.92	CheA Cj0284c
1.17	1.14	1.16	1.12	1.15	0.91	0.98	0.82	0.91	CheV Cj0285c
1.13	1.06	1.10	1.01	1.08	0.99	1.00	0.72	0.90	CheW Cj0283c
0.95	0.78	0.91	0.84	0.87	0.85	0.78	0.98	0.87	Aer1 CetC Cj1191c
0.82	0.86	0.78	0.86	0.83	0.83	0.96	0.75	0.84	Tlp1 CcaA Cj1506c
0.65	0.72	0.66	0.63	0.66	0.88	0.74	0.68	0.77	Tlp9 CetA Cj1190c
0.77	0.80	0.81	0.67	0.76	0.66	0.83	0.71	0.73	Tlp3 CcmL Cj1564
0.75	0.71	0.76	0.71	0.73	0.73	0.69	0.71	0.71	Aer2 CetB Cj1189c
0.31	0.71	0.73	0.48	0.56	0.59	0.67	0.53	0.60	Tlp8 CetZ Cj1110c

B

5% 1	5% 2	5% 3	5% 4	5% Mean	100% 1	100% 2	100% 3	100% Mean	
0.90	1.78	1.00	1.87	1.39	1.53	2.87	2.58	2.32	MurF Cj0795c
1.70	1.74	1.17	1.48	1.52	2.59	2.03	1.54	2.05	MurE Cj1641
1.35	1.38	1.82	2.03	1.65	2.29	1.58	2.02	1.96	MurD Cj0432c
						1.80		1.80	MltD Cj0645
1.52	1.05	1.19	1.45	1.30	1.86	1.45	1.97	1.76	MltG Cj0529c
1.03	1.43	1.51	1.95	1.48	1.51	1.34	2.18	1.68	CslT Cj0843c
1.32	1.76	1.24	1.87	1.55	1.74	1.29	1.97	1.67	PatA Cj0610c
1.37	1.38	1.36	1.57	1.42	2.12	1.56	1.27	1.65	RlpA Cj0646
		0.90	1.09	1.00		1.59	1.65	1.62	MraY Cj0433c
1.80	1.27	1.02	1.16	1.31	1.89	1.46	1.50	1.61	Csd1 Cj1087c
1.49	1.65	1.14	1.45	1.44	1.82	1.30	1.44	1.52	PbpC Cj0652
1.14	1.83	1.01	1.25	1.31	1.38	1.20	1.43	1.34	PbpB Cj0525c
1.36	1.27	1.31	1.18	1.28	1.43	1.18	1.24	1.29	MurC Cj1054c
1.19	1.18	1.35	1.34	1.27	1.27	1.27	1.09	1.21	MurI Cj1652c
0.99	1.02	0.95	0.98	0.99	1.91	0.77	0.86	1.18	DdlA Cj0798c
1.00	0.91	0.85	0.86	0.91	1.79	0.87	0.67	1.11	CcmA Cj1086c
0.85	0.82	0.90	0.92	0.87	1.40	1.12	0.77	1.10	MreC Cj0277
0.91	0.81	0.79	0.88	0.85	0.88	1.00	1.31	1.06	UppS Cj0824
1.10	0.97	1.15	1.06	1.07	0.81	1.33	1.03	1.06	MurG Cj1039
0.79	0.90	0.80	0.92	0.85	0.83	1.06	1.27	1.05	AmiA Cj1269c
0.96	1.01	1.00	1.03	1.00	0.95	0.99	1.11	1.02	MurA Cj0858c
0.82	0.82	0.99	0.88	0.88	0.94	0.94	1.15	1.01	MurB Cj1676
1.00	1.03	1.07	1.08	1.04	1.52	0.77	0.69	0.99	MreB Cj0276
0.61	0.72	0.71	0.78	0.70	1.05	1.23	0.68	0.99	Csd3 Cj1215
0.65	0.70	0.66	0.74	0.69	0.99	0.94	0.97	0.97	Cj0131
0.87	0.88	0.87	0.88	0.88	0.76	1.25	0.79	0.93	PbpA Cj0508
0.96	0.92	0.96	0.99	0.96	0.68	0.98	1.15	0.93	Cj1275c
0.88	0.89	0.86	0.99	0.90	0.70	0.94	0.91	0.85	Ape1 Cj0609c
0.71	0.76	0.75	0.79	0.75	0.71	0.90	0.88	0.83	Pgp2 Cj0906c
0.74	0.77	0.68	0.80	0.75	1.08	0.81	0.52	0.80	Pgp3 Cj1235

0.4 1.0 2.5

Supplementary Figure S5. Growth in CE results in changes to abundances of proteins involved in chemotaxis and peptidoglycan (PGN) biosynthesis, modification and cell shape. Heat map showing replicate (MH+5% CE [left; *n*=4] and 100% CE [right; *n*=3] compared with MH only control) and mean *n*-fold data for proteins involved in (**A**) chemotaxis and (**B**) PGN biosynthesis, modification and cell shape.

A

5% 1	5% 2	5% 3	5% 4	5% Mean	100% 1	100% 2	100% 3	100% Mean	
1.33	1.26	3.02	2.02	1.91	5.14	2.31	1.65	3.03	PglD Cj1123c
1.07	2.34	1.16	1.50	1.52	3.84	1.52	1.28	2.21	PglK WlaB Cj1130c
2.46	1.29	1.55	1.79	1.77	2.54	2.32	1.71	2.19	PglH Cj1129c
0.93	1.53	4.75	0.91	2.03	0.89	3.96	1.34	2.06	PglC Cj1124c
1.18	1.24	1.57	1.10	1.27	1.72	2.50	1.63	1.95	PglA Cj1125c
1.03	1.70	1.76	1.11	1.40	1.41	1.80	1.87	1.69	PglJ Cj1127c
1.28	1.24	1.31	1.41	1.31	1.29	2.27	1.30	1.62	PglI Cj1128c
2.19	0.85	1.27	1.02	1.33	1.32	1.96	1.35	1.54	PglB Cj1126c
1.06	1.38	1.22	1.23	1.22	1.40	1.11	1.28	1.26	PglF Cj1120c
1.05	1.09	0.98	1.14	1.07	1.16	1.27	1.19	1.21	PglE Cj1121c
0.80	0.73	0.96	0.93	0.86		1.13	0.42	0.78	PglG Cj1119c

0.4 1.0 2.5

B

5% 1	5% 2	5% 3	5% 4	5% Mean	100% 1	100% 2	100% 3	100% Mean	
1.25	3.54	2.14	3.72	2.66	4.84	2.27	3.12	3.41	Cj1661
0.71	0.60	0.72	0.66	0.67	2.85	2.05	4.50	3.13	Cj0011c
1.58	1.42	1.40	2.26	1.66		3.27	2.87	3.07	CtsD Cj1474c
1.12	1.33	2.11	3.58	2.04		2.88	3.00	2.94	Cj0177
0.92	1.17	0.91	1.12	1.03	2.59	2.73	3.18	2.84	Cj0114
3.28	2.32	1.19	1.18	1.99	2.91	3.00	1.75	2.55	Cj0182
1.98	1.81	2.21	1.39	1.85	2.71	1.73	2.97	2.47	Cj0608
2.33	1.28	2.41	2.30	2.08	2.91	1.78	1.99	2.23	Cj0397c
0.94	0.95	1.01	1.06	0.99	3.01	1.53	1.87	2.14	RplQ Cj1596
1.10	1.11	1.26	1.86	1.33	2.80	2.02	1.41	2.08	CmeA AcrA Cj0367c
0.86	0.88	0.69	0.71	0.79	2.06	2.22	1.66	1.98	Cj0515
1.64	1.56	1.40	2.31	1.73	2.55	1.89	1.45	1.96	CgpA Cj1670c
1.55	1.44	1.33	1.33	1.41	2.68	1.24		1.96	Cj0540
1.03	1.44	1.26	2.87	1.65	2.71	1.24	1.86	1.94	Cj0648
1.63	1.34	1.72	1.32	1.50	1.64	2.73	1.06	1.81	Cj1496c
0.89	1.13	1.80	1.39	1.30	2.31	1.58	1.49	1.80	FlgP Cj1026c
1.34	1.19	1.07	1.82	1.35	1.60	1.52	2.22	1.78	Cj0200c
1.87	2.05	0.97	1.18	1.52	1.93	1.87	1.42	1.74	Cj0911
1.91	1.82	1.51	1.55	1.70	1.91	1.51	1.67	1.70	Cj0199c
1.37	1.32	1.42	1.52	1.41	1.29	2.07	1.70	1.69	CmeE Cj1032
1.03	1.43	1.51	1.95	1.48	1.51	1.34	2.18	1.68	Cj0843c
1.32	1.76	1.24	1.87	1.55	1.74	1.29	1.97	1.67	PatA Cj0610c
1.75	2.25	1.20	1.39	1.65	2.31	1.38	1.23	1.64	Cj0313
1.15	2.14	1.52	1.51	1.58	1.54	1.51	1.66	1.57	Cj0941c
1.83	1.99	1.42	1.26	1.63	1.57	1.47	1.61	1.55	Cj1373
1.04	1.20	1.21	1.34	1.20	1.46	1.65	1.53	1.55	CmeC Cj0365c
2.19	0.85	1.27	1.02	1.33	1.32	1.96	1.35	1.54	PglB Cj1126c
1.49	1.65	1.14	1.45	1.44	1.82	1.30	1.44	1.52	PbpC Cj0652
0.86	0.88	1.01	1.18	0.99	1.62	1.28	1.53	1.48	DsbI Cj0017c
1.10	1.37	1.32	1.27	1.26	1.72	1.47	1.02	1.40	NapB Cj0783
1.34	1.13	1.27	1.07	1.20	1.36	1.36	1.42	1.38	CmeB Cj0366c
1.42	1.41	1.55	1.46	1.46	1.32	1.59	1.06	1.32	PyrG Cj0027
1.23	1.23	1.10	1.21	1.19	1.09	1.40	1.40	1.30	Cj0944c
1.10	1.02	1.04	1.01	1.04	1.49	1.22	1.10	1.27	PflA Cj1565c
0.80	0.89	0.77	0.83	0.82	1.54	1.09	1.03	1.22	Cj1207c
1.10	1.06	1.11	1.06	1.08	1.75	0.87	0.94	1.19	Cj0376
0.95	0.93	0.97	0.98	0.96	1.27	1.11	1.14	1.17	Cj0694
1.04	1.21	0.93	1.02	1.05		2.41	1.17	1.17	Cj0455c
0.87	0.80	0.72	0.73	0.78	1.68	0.72	1.00	1.13	Cj0846
0.86	0.74	0.69	0.70	0.75	1.27	0.91	1.21	1.13	Cj0238
0.89	1.06	1.04	0.99	1.00	1.20	0.99	1.17	1.12	Cj0399
0.93	0.85	1.18	1.17	1.03	1.23	1.12	0.98	1.11	Cj0267c
0.85	0.82	0.90	0.92	0.87	1.40	1.12	0.77	1.10	MreC Cj0277
0.87	0.90	0.88	0.88	0.88	1.09	1.05	1.11	1.08	Cj0530
0.87	0.86	0.79	0.80	0.83	1.29	0.99	0.96	1.08	EptC Cj0256
1.13	1.10	1.05	1.01	1.07	0.71	0.97	1.49	1.06	Cj0497
1.26	1.15	1.31	1.17	1.22	0.96	0.98	1.15	1.03	NapA Cj0780
1.25	1.14	1.16	1.10	1.16	1.36	0.87	0.81	1.01	KpsD Cj1444c
0.89	0.85	0.80	0.96	0.88	1.20	0.85	0.99	1.01	PflB Cj0390
0.86	0.85	0.85	0.86	0.86	0.98	1.19	0.84	1.00	Cj1219c
0.79	0.89	0.87	0.88	0.86	0.98	0.92	1.10	1.00	YidC Cj0958c
0.99	0.89	1.01	1.21	1.02	1.05	0.96	0.97	1.00	Cj0633
1.42	1.51	1.18	1.20	1.32	0.80	1.44	0.72	0.99	Cj0158c
0.87	0.87	0.79	0.79	0.83	1.06	1.05	0.81	0.97	Cj0371
0.65	0.70	0.66	0.74	0.69	0.99	0.94	0.97	0.97	Cj0131
1.05	1.08	1.02	1.03	1.04	1.08	0.94	0.88	0.97	UvrA Cj0342c
0.86	0.87	0.85	0.78	0.84	0.93	0.90	1.01	0.95	Cj1055c
0.87	0.88	0.87	0.88	0.88	0.76	1.25	0.79	0.93	PbpA Cj0508
0.88	0.97	0.67	0.80	0.83	1.04	0.87	0.85	0.92	Cj1007c
0.86	0.85	0.86	0.85	0.85	0.64	1.04	1.05	0.91	Cj1621
1.14	1.06	1.06	1.03	1.07	0.95	0.84	0.94	0.91	Cj0511
0.89	0.86	0.74	0.86	0.84	0.71	0.77	1.20	0.90	Cj0599
0.80	0.81	0.83	0.90	0.83	0.88	0.92	0.87	0.89	Cj1433c
1.07	1.02	1.15	1.03	1.07	0.76	0.84	1.00	0.87	FabF Cj0442
0.66	0.73	0.68	0.67	0.69	0.81	0.94	0.82	0.86	Cj0268c
0.84	0.82	0.91	0.91	0.87	0.98	0.85	0.74	0.86	Cj0152c
0.49	0.69	0.62	0.70	0.62	0.91	0.89	0.69	0.83	Rp10 Cj1689c
0.77	0.79	0.73	0.72	0.75	0.66	0.87	0.97	0.83	LegI NeuB2 Cj1327
0.71	0.76	0.75	0.79	0.75	0.71	0.90	0.88	0.83	Pgp2 Cj0906c
0.69	0.73	0.77	0.69	0.72	0.78	0.70	0.99	0.82	Cj0776c
1.11	1.03	0.77	0.64	0.89	1.11	0.78	0.56	0.82	Cj0089
0.68	0.65	0.67	0.59	0.65	0.56	0.55	1.24	0.79	CioA CydA Cj0081
0.53	0.79	0.52	0.74	0.64	0.79	0.75	0.80	0.78	JlpA Cj0983
0.83	0.76	0.67	0.76	0.76	0.66	0.60	1.05	0.77	Cj0591c
0.26	0.35	0.50	0.49	0.40		0.72	0.72	0.72	Cj0168c
0.70	0.80	0.77	0.79	0.77	0.86	0.66	0.52	0.68	Cj0404
0.85	0.53	0.43	0.78	0.65	0.84	0.47	0.56	0.62	ZnuA Cj0143c
0.72	0.78	0.66	0.72	0.72	0.13	0.75	0.95	0.61	Cj1013c
0.78	0.83	0.76	0.86	0.81		0.70	0.52	0.61	Cj0454c
0.65	0.54	0.64	0.62	0.61	0.63	0.71	0.46	0.60	Cj0592c
0.85	0.28	0.36	0.45	0.48	0.85	0.46	0.41	0.57	HisJ CjaC Cj0734c
0.45	0.42	0.48	0.40	0.43	0.33	0.59	0.54	0.49	CjaA Cj0982c
0.91	0.86	0.26	0.27	0.58	0.42	0.47		0.45	Cj0494
0.52	0.52	0.49	0.15	0.42	0.25	0.43	0.52	0.40	Peb3 Cj0289c
1.02	1.43	0.93	1.20	1.14					SecG Cj0235c

C

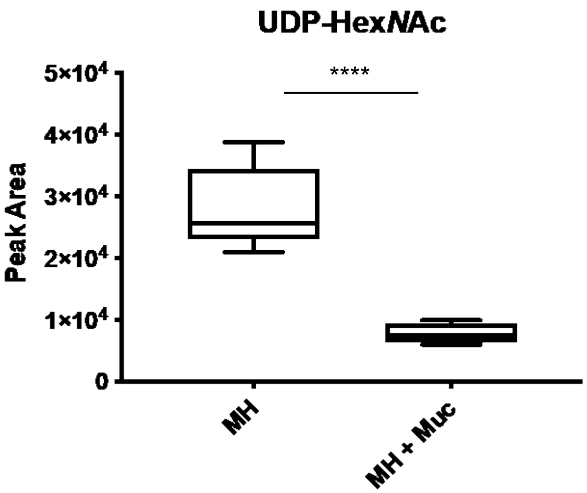

Supplementary Figure S6. Growth in CE results in increased abundances of proteins associated with the Pgl *N*-linked glycosylation system. Heat maps showing replicate (MH+5% CE [left; n=4] and 100% CE [right; n=3] compared with MH only control) and mean n-fold data for proteins involved in (**A**) biosynthesis and attachment of *N*-linked glycans to proteins (Pgl cluster); (**B**) known targets of *N*-linked glycosylation; (**C**) intracellular levels of UDP-Hex*N*Ac as determined by targeted intracellular metabolomics, ****, $p < 0.0001$.
