# Supplementary material for: Multi-Omics of Campylobacter jejuni Growth in Chicken Exudate Reveals Molecular Remodelling Associated with Altered Virulence and Survival Phenotypes
Source: Microorganisms. 2024 Apr 25;12(5):860. doi: 10.3390/microorganisms12050860 (PMC11123243; doi:10.3390/microorganisms12050860)
Supplement: Supplementary file 1 [file microorganisms-12-00860-s001.zip › Supplementary Tables S1_S2.pdf]

Supplementary Table S1. Instrument settings for targeted metabolomics using LC-MS/MS. Q1, precursor *m/z*; Q3, selected fragment ion *m/z*; Dwell (ms), dwell time on precursor; CE, collision energy (volts); CXP, collision cell exit potential (volts); DP, declustering potential (volts).

Synergi-RP Negative Mode MRM

Name	Q1	Q3	Dwell (ms)	CE	CXP	DP
Hexose (Glu/Fuc/Gal)	179	32	100	-26	-15	-45
Lactate	89	43	100	-17	-11	-34
Citrate	191	111	100	-19	-12	-50
Fumarate	115	71	100	-16	-9	-35
Succinate	117	73	100	-24	-10	-24
UDP-HexNAc	606	79	100	-128	-9	-140

Luna Phenyl-Hexyl MRM

Name	Q1	Q3	Dwell (ms)	CE	CXP	DP
Anserine	241	109.2	100	35	16	31
Histidine	156	110	100	21	12	49
Carnosine	227	110	100	33	14	51
Carnitine	162.1	60	100	23	8	41
Acetyl-carnitine	204	85	100	31	16	46
Homocysteine	136	119	100	25	14	101
Leucine/Isoleucine	132.1	86.2	100	16	22	37
Lysine	147.1	84.1	100	25	12	51
Glutamate	148	84	100	23	10	54
Glutamine	147	44	100	73	10	51
Asparagine	133.1	74	100	22	10	56
Proline	116	70	100	13	13	51
Methionine	150	61	100	44	10	44
Serine	106	88	100	15	10	46
Alanine	90.1	44.1	100	16	8	41
Homoserine	120	44	100	17	10	51
Glycine	76	30	100	9	13	40
Tryptophan	205.1	188.3	100	15	12	43
Valine	118	72	100	16	15	61
Threonine	120.1	74	100	15	8	44
Cysteine	122	59	100	26	9	63
Cystine	241	152	100	35	15	40
Aspartate	134	74	100	20	13	85
Arginine	175.1	70	100	32	10	25

Supplementary Table S2. Quantitative proteomics of growth in 5% and 100% CE by TMT label-based shotgun proteomics displays profound changes in the *C. jejuni* NCTC11168 proteome at different stringencies. Proteins were defined as altered in abundance at +/-1.5-fold mean *n*-fold change (<0.67 and >1.5-fold), when observed in 2 of 4 (5% CE) or 2 of 3 (100% CE) biological replicates and with $p<0.05$. Data for all identified (1386 and 1326 proteins for 5% and 100% CE comparisons with MH controls, respectively) and quantified (1328 and 1304 proteins, respectively) proteins can be found in Data S1.

Growth Comparison	⇑ @ 1.5-fold	⇑ @ 1.5-fold in 2 expt.	⇑ @ 1.5-fold in 2 expt. and $p<0.05$	⇓ @ 0.67-fold	⇓ @ 0.67-fold in 2 expt.	⇓ @ 0.67-fold in 2 expt. and $p<0.05$
**5% CE**	136	107	79	98	88	74
**100% CE**	277	214	207	105	83	78
